# Dysfunctional labor and hemoperitoneum secondary to an incidentally discovered dysgerminoma: a case report

**DOI:** 10.1186/s12884-021-04063-2

**Published:** 2021-09-07

**Authors:** Aneesa Thannickal, Brandon Maddy, Marla DeWitt, William Cliby, Margaret Dow

**Affiliations:** 1 Department of Obstetrics and Gynecology, Mayo Clinic, 200 1st St SW, Rochester, MN 55905 USA; 2 Department of Family Medicine, Mayo Clinic, Rochester, MN USA; 3 Division of Gynecology Oncology, Mayo Clinic, Rochester, MN USA

**Keywords:** Dysgerminoma, Pregnancy, Protracted labor, Hemoperitoneum, Oophorectomy, Staging, Case report

## Abstract

**Background:**

Ovarian dysgerminoma, a subtype of malignant germ cell tumor (GCT), is a rare ovarian neoplasm that is infrequently found in the gravid patient. When dysgerminomas do occur in pregnancy, the rapidly growing tumors can have a heterogeneous presentation and lead to peripartum complications and morbidity. Due to the rarity of this condition, diagnostic and therapeutic strategies are not well described in the literature.

**Case presentation:**

A healthy multigravida with an uncomplicated antenatal history presented for elective induction of labor. She had a protracted labor course, persistently abnormal cervical examinations, and eventually developed a worsening Category II tracing that prompted cesarean birth. Intraoperatively, a 26 cm pelvic mass later identified as a Stage IA dysgerminoma was discovered along with a massive hemoperitoneum. The mass was successfully resected, and the patient remains without recurrence 6 months postoperatively.

**Conclusion:**

Although rare and generally indolent, dysgerminomas can grow rapidly and cause mechanical obstruction of labor and other complications in pregnancy. Pelvic masses, including malignant neoplasms, should be included in as part of a broad differential diagnosis when evaluating even routine intrapartum complications such as abnormal labor progression. Additionally, we demonstrate that adnexal masses can be a source of life-threatening intraabdominal hemorrhage.

## Background

Malignant ovarian neoplasms rarely occur during pregnancy, with an estimated incidence of 2.8–11 per 100,000 pregnancies [1–3]. Dysgerminomas represent a relatively large portion of these cancers diagnosed in pregnancy, although absolute incidence is very low [4, 5]. Prior reviews of existing case reports on this entity have demonstrated that dysgerminoma in pregnancy is typically discovered early during routine antenatal imaging or secondary to symptoms that develop prior to labor [4]. Cases that describe incidental discovery of a dysgerminoma at the time of delivery tend to describe patients in low resource settings, with inadequate prenatal care, or who delivered prior to the routine adoption of routine antenatal ultrasonography [4, 6, 7]. Here, we present a unique case where a large dysgerminoma went undiagnosed until labor in a patient who otherwise had an uncomplicated prenatal course with adequate antepartum care. The patient went on to develop a labor dystocia, which has been rarely described in the literature [7, 8] as well as a massive hemoperitoneum, which has not been reported. In this report, we will review the diagnosis and management of this rare pregnancy complication.

## Case presentation

A previously healthy Caucasian 32-year-old gravida two, para one was admitted to our obstetrics service at 40 weeks gestational age (GA) for elective induction of labor. Her pregnancy was complicated by mild anemia diagnosed in the second trimester, chronic constipation that worsened around 36 weeks GA, and excessive weight gain of 17.3 kg (upper limit of normal for this patient’s BMI is 16 kg). Her medical, obstetrical, gynecological and surgical history were non-contributory.

**Fig. 1:**
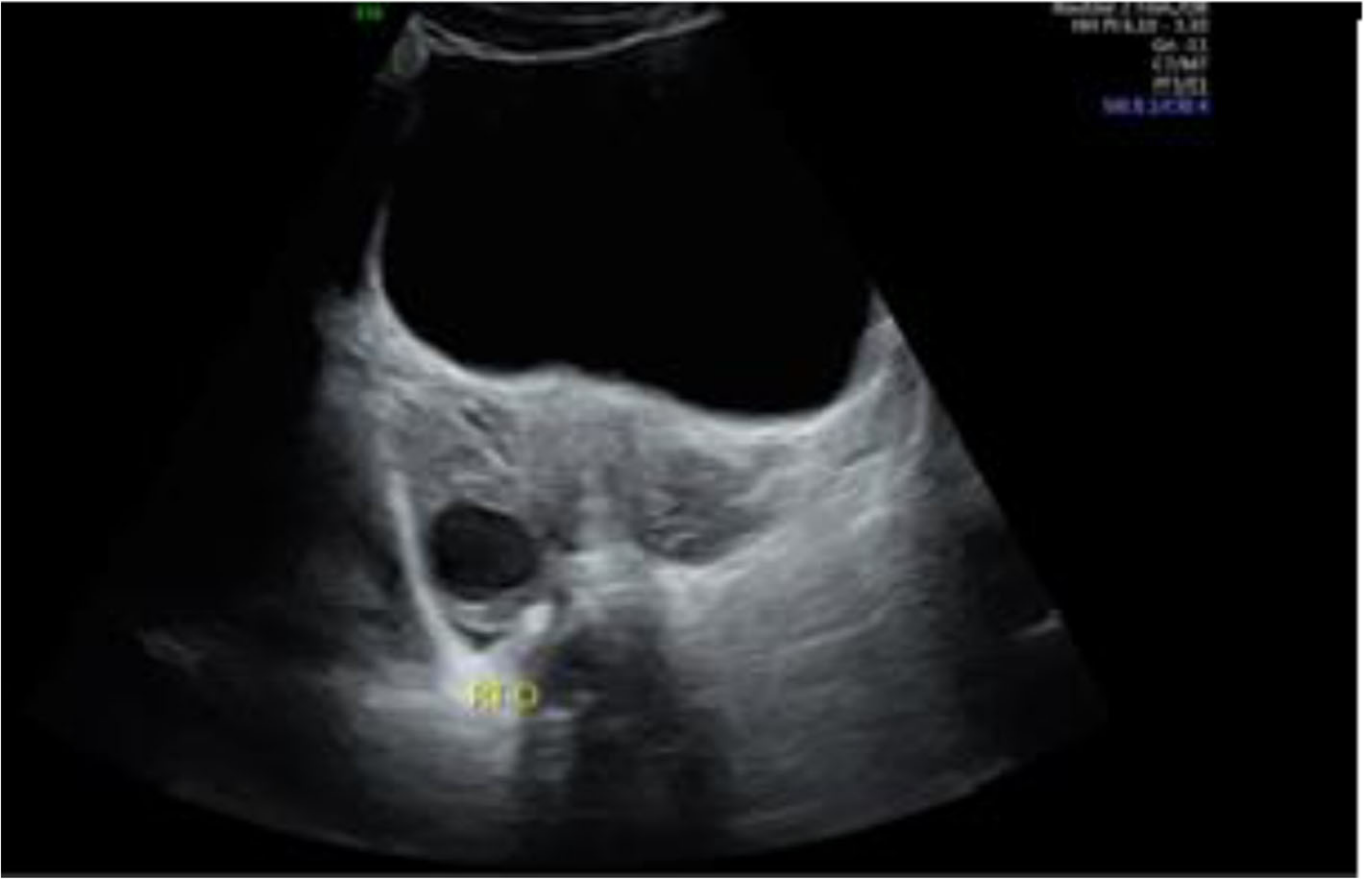
First trimester US performed at 9w1d GA. Right ovary demonstrated a cystic mass which was read initially as normal post-ovulatory change

**Fig. 2:**
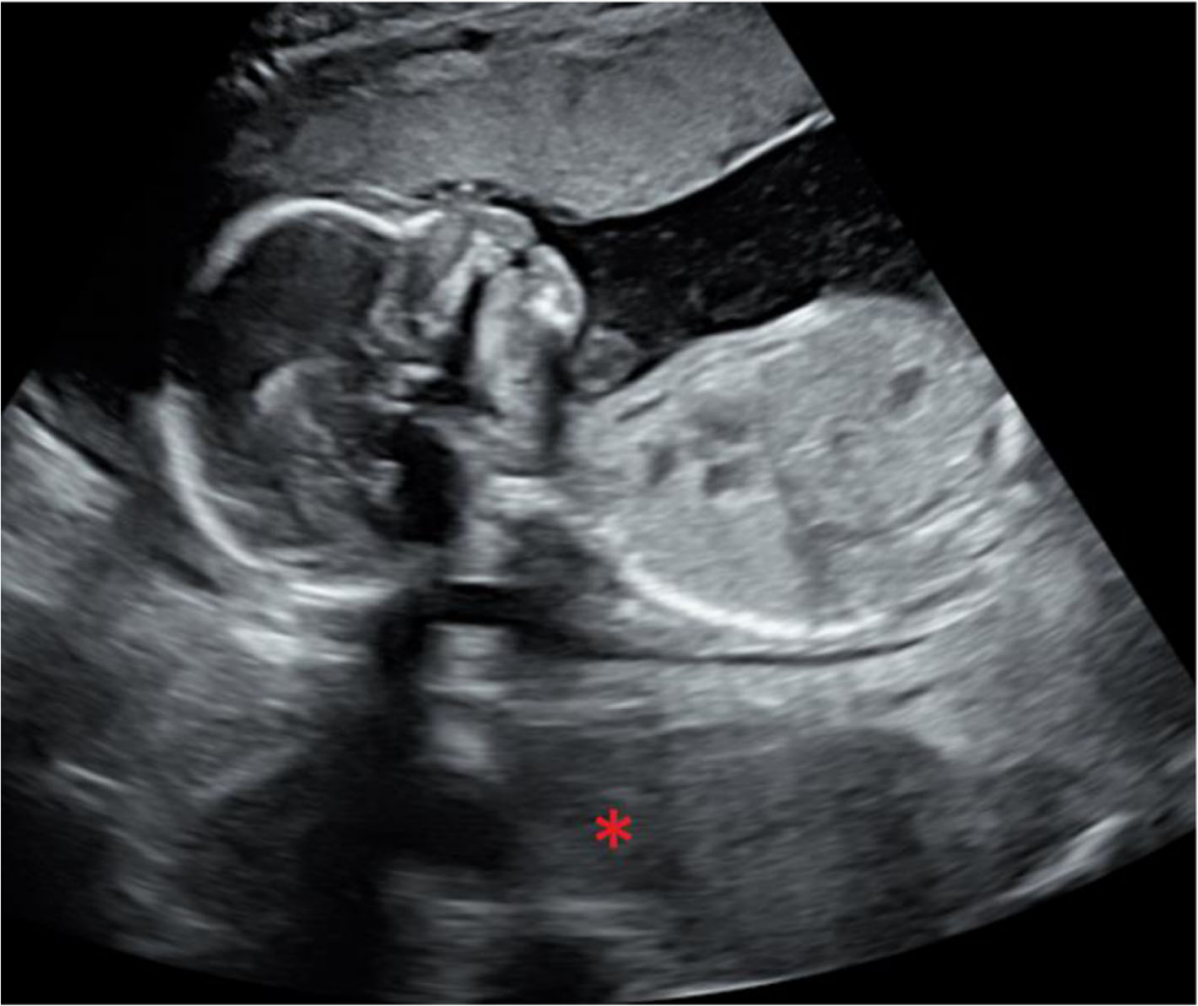
Sagittal image from anatomy scan performed at 18w4d GA. A large, hypoechoic region can be visualized posterior to the uterus, attributed to bowel gas at the time of imaging (indicated by asterisk)

She underwent a transabdominal ultrasound (TAUS) at 9w1d GA that showed fetal growth consistent with GA. Both ovaries were visualized and determined to be normal appearing with a post-ovulatory-appearing right ovary (Fig. 1). Standard anatomy scan at 19w1d showed a fetus with bilateral choroid plexus cysts but no other abnormalities. Adnexae were unable to be completely visualized. (Fig. 2). Her palpated fundal height throughout the pregnancy remained within normal limits; therefore, she did not undergo any additional imaging per our institutional standard.

On admission, the patient was well-appearing with normal vital signs. Fetal heart tracing (FHT) was reactive. Lab studies obtained on admission were notable for a hemoglobin of 8.6 g/dL and platelet count of 131 × 10^9^ /L. Cervical exam was extremely difficult initially, with the cervix located anterior and shifted toward the patient’s left. Fetal station was −2. Her induction began with vaginal misoprostol followed by mechanical dilation with a Foley balloon. After Foley expulsion, the fetal station persisted at −2 despite a uterine contraction frequency of 1–3 min. An epidural catheter was placed for neuraxial anesthesia. Artificial rupture of membranes was deemed inappropriate due to disengagement of the fetal head. Intravenous (IV) Pitocin was initiated around 15 h after admission. Soon after initiation of oxytocin, she developed a fever (T_max_ 38.0 °C), right upper quadrant pain, as well as an intermittent Category II FHT with late decelerations. An evaluation for fever of unknown origin was initiated. Lab studies were obtained at this time and notable for large blood on urinalysis, 51–100 white blood cells on urine microscopy, hemoglobin of 7.8 g/dL and platelet count of 118 × 10^9 /L. Lactate dehydrogenase (LDH), which was obtained due to concern for atypical HELLP syndrome (hemolysis, elevated liver enzymes, and low platelets), was markedly elevated at 1518 U/L. AST was 78 U/L, and total bilirubin was normal at 0.3 mg/dL. In addition to atypical HELLP syndrome, urinary tract infection (UTI) and chorioamnionitis were considered as diagnoses. One dose of ceftriaxone was administered for suspected UTI before the decision was made to broaden antimicrobial coverage to ampicillin and gentamicin such that any possible intraamniotic infection was treated.

**Fig. 3:**
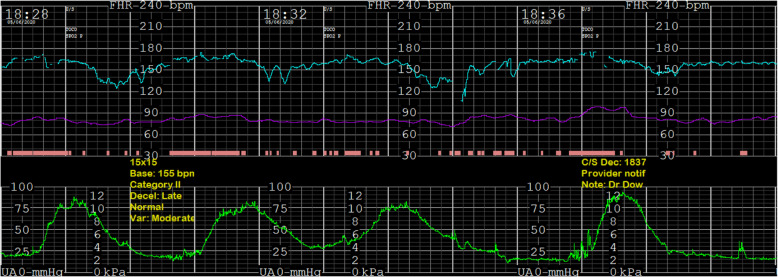
FHT about 20 min prior to proceeding with cesarean delivery. Tracing is Category 2, with a baseline of 160 beats per minute, moderate variability, no accelerations, and persistent late decelerations

At hour 25 of induction, a bedside TAUS was performed due to the unusual and unchanged cervical examination characteristics. It demonstrated a fetus in cephalic presentation as well as a poorly defined mass in the posterior cul-de-sac without flow on color Doppler imaging. The differential diagnosis was broadened to include adnexal mass, cervical fibroid or stool from ongoing constipation. At this point, the FHT deteriorated to persistent Category II with repetitive late decelerations and moderate variability with a normal baseline (Fig. 3), necessitating primary cesarean birth for non-reassuring fetal status remote from delivery.

A primary cesarean delivery via Pfannenstiel incision was performed resulting in the delivery of a vigorous female infant with a weight of 3030 g. Hemoperitoneum was identified upon abdominal entry. After delivery of the placenta and hysterotomy closure, uterine atony was noted requiring the use of multiple uterotonic agents. Once uterine hemostasis was achieved, the pelvis was explored.

Arising from the right ovary was a solid fibrous mass with associated bleeding from a vascular pedicle. A gynecologic oncologist was enlisted for the procedure. The patient was placed under general anesthesia and an abdomino-pelvic survey was performed. There were no visual signs of metastatic disease on the pelvic peritoneum, viscera, or infracolic omentum and no palpable masses in the upper abdomen. A right salpingo-oophorectomy was then performed as well as biopsies of the posterior cul-de-sac and left ovarian surface. Given the limited availability of frozen pathology, concern for provoking disseminated intravascular coagulopathy, and the uncertain nature of the mass, the procedure was then concluded. Specimens were sent for permanent pathology. The patient’s quantitative blood loss was 4.3 L by the conclusion of the procedure, with 1.5 L attributed to preexisting hemoperitoneum and 2.8 L due to bleeding from uterine atony and continued bleeding from the vascular pedicle of the tumor. She received two units of packed red blood cells (PRBC) intraoperatively. Massive transfusion protocol was not initiated as bedside point-of-care hemoglobin measurements and serial intraoperative measures of prothrombin time, activated partial thromboplastin time, and fibrinogen remained stable.

Postoperatively, the patient was placed on ampicillin, gentamicin, and clindamycin for 24 h given her previous fever of unknown origin, and she remained afebrile for the rest of her hospital course. Postoperative alpha fetoprotein (AFP) and inhibin A and B were obtained and were within the reference range (56 ng/mL, 25 pg/mL, and < 10 pg/mL, respectively). She required a third unit of PRBCs on postoperative day one for symptomatic anemia but had an otherwise uncomplicated postoperative course. Her neonate suffered no apparent birth complications, and both were dismissed to home without prolonged hospitalization. She was subsequently evaluated in the outpatient setting for postpartum endometritis and was treated with oral amoxicillin/clavulanate.

**Fig. 4:**
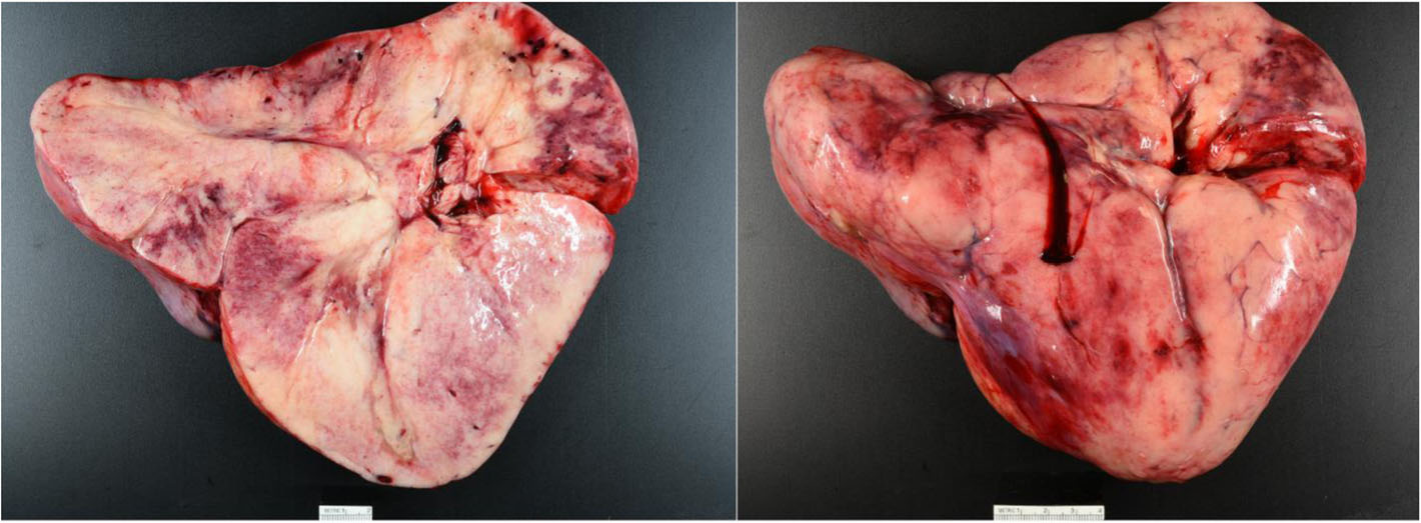
Gross pathology image of resected dysgerminoma. Mass weighed 1564 g and dimensions were 26 × 18 × 9 cm. Pale tan-pink, lobulated, well-circumscribed outer surface is typical in appearance for dysgerminomas. The cut surface of the mass is mostly homogeneous solid tissue with focal areas of hemorrhage

**Fig. 5:**
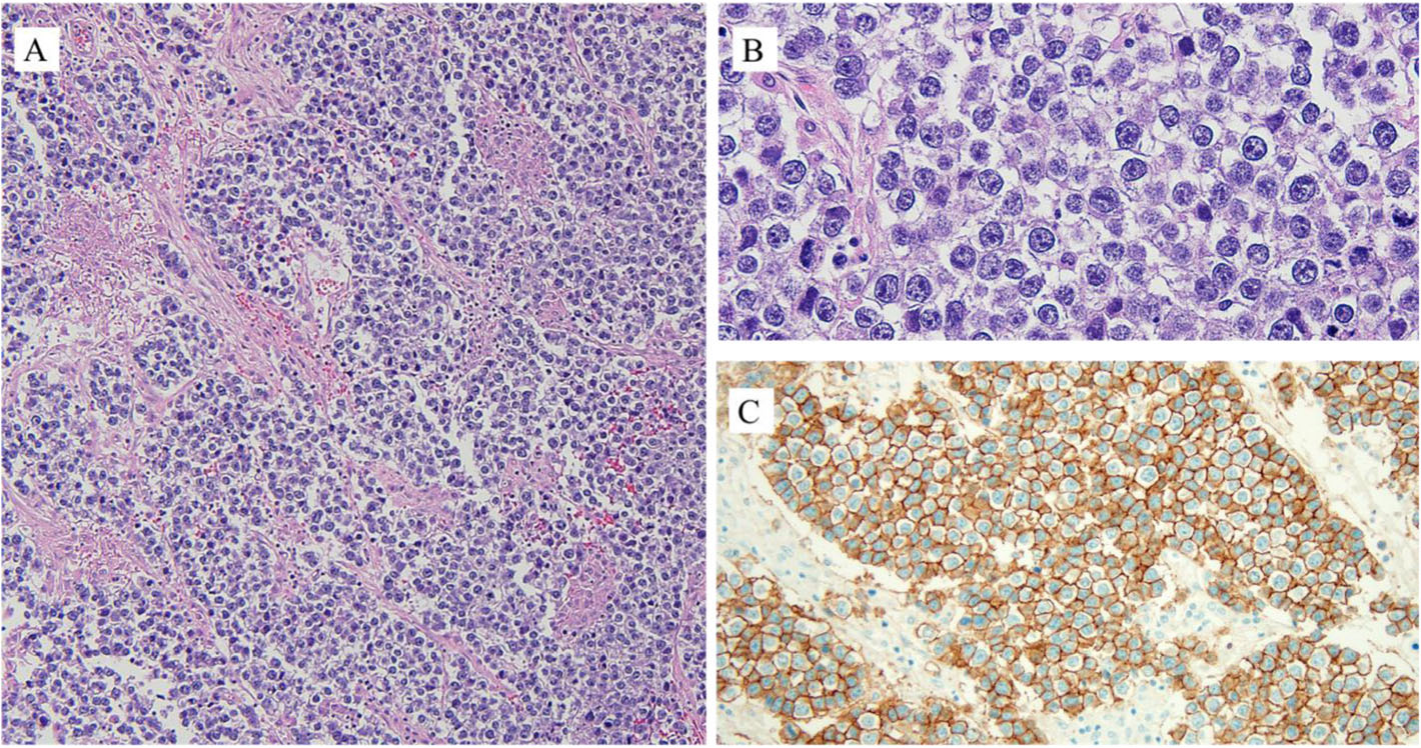
Dysgerminoma shows nested growth of uniform polygonal cells with clear to partial eosinophilic cytoplasm, angulated to ovoid nuclei and an associated lymphocytic infiltrate (**A**). Under higher magnification (**B**). The tumor is positive for OCT3/4 (cytoplasmic and nuclear), SALL4 (nuclear) and KIT (membranous and cytoplasmic) (**C**)

Final pathology of the mass demonstrated a 26 × 18 × 9 cm solid mass with histologic features consistent with a dysgerminoma (Figs. 4 and 5). All biopsies were negative for tumor. The patient underwent CT imaging of the chest, abdomen and pelvis on postoperative day seven which was negative for signs of metastatic disease. The final tumor stage was assigned IA and the patient received no adjuvant therapy. She entered a period of surveillance with good adherence to care and serial follow up visits. LDH on postoperative day seven was 420 U/L, and approximately 1 month later had returned to normal levels at 190 U/L. At 6 months, she is without evidence of disease.

## Discussion and conclusions

Here we present a rare case of an ovarian dysgerminoma incidentally discovered at the time of delivery. While other case reports of similar entities exist dating back to the early twentieth century, our case serves as an interesting learning point for several reasons: our patient had adequate prenatal care and imaging documenting a normal appearing right ovary in the first trimester, suggesting that this tumor developed de novo antepartum; she remained asymptomatic from the massive tumor through most of her pregnancy; she had a labor dystocia that is most likely attributable to mass effect or incarceration of the tumor; and she developed a clinically significant hemoperitoneum as a result of trauma to the vascular supply to the mass.

Reproductive age women tend to be at relatively higher risk for germ cell tumors, both benign and malignant. Dysgerminomas, a subtype of malignant germ cell tumor, are one of the most uncommon ovarian neoplasms. The incidence is roughly 0.1–1 cases per 100,000 pregnancies [1]. As such, there are few recommendations regarding diagnosis and management of this neoplasm in the gravid patient. Exacerbating this issue is the lack of prospective randomized treatment studies and therefore objective data to establish clinical guidelines for any pregnancy-associated ovarian cancers [2, 9].

Pregnancies associated with ovarian malignancies require balancing optimal maternal therapy and fetal well-being. In addition, cancer diagnosis may be delayed because of difficulties in distinguishing symptomatology from physiologic changes in pregnancy [4, 10]. The symptoms of a dysgerminoma outside of pregnancy tend to be non-specific and include pelvic fullness, early satiety, urinary frequency, dysuria and constipation [4], all of which can also be observed in the course of a normal pregnancy. In the presented case, the patient was asymptomatic from her right sided ovarian mass for the majority of her pregnancy with worsening constipation being the only notable symptom. Similarly, tumor markers commonly associated with malignant germ cell tumors (human chorionic gonadotropin and AFP in particular) are elevated during pregnancy, which can lessen their diagnostic value [5, 11]. In our case, a significantly elevated LDH was identified during her laboratory evaluation but was preferentially attributed to developing HELLP syndrome over a dysgerminoma.

Sonography is crucial in assessing adnexal masses in the first trimester. Technicians will routinely assess the adnexa for corpus luteal cysts, rare heterotopic pregnancy and ovarian and tubal pathology. The overall incidence of adnexal masses ranges from 2 to 10% in the first trimester [10, 12]. Most masses seen in the first trimester spontaneously regress with advancing gestational age [12]. In review of this patient’s first trimester ultrasound and anatomy scan the right ovary was sub-optimally visualized in the second trimester, preventing any definitive impressions. As in our patient’s case, if suboptimal visualization is reported, it is usually due to position of the ovaries behind the gravid uterus [13]. Additionally, the uniformly solid makeup of dysgerminomas can make diagnosis on ultrasound challenging compared to other malignancies which have a mixed solid-cystic structure.

Overall, the effects of pregnancy on dysgerminomas are not well studied. Similar to other adnexal masses, there is increased risk of torsion, incarceration, rupture and hemorrhage that can occur during pregnancy. Intrauterine growth restriction was the most commonly identified adverse fetal outcome in one case series [4]. Our case demonstrates that labor dystocia may be a presenting symptom of a massive dysgerminoma. Key clinical findings included abnormal cervical exams and a dysfunctional labor curve. The patient’s cervix was found to be extremely anterior and above the pubic symphysis and continued to stay in this position for greater than 24 h. Additionally, she made minimal cervical change despite efforts at labor augmentation, an unexpected finding in a multigravida. In retrospect, we believe that the very large dysgerminoma acted to antevert the uterus and caused a physical obstruction which prevented fetal descent and active cervical dilation. As such, this represents one of the few reported cases of ovarian dysgerminoma associated with a labor dystocia [4, 14–16]. This mechanical obstruction theory has been explored in the literature regarding leiomyomas, however data on causation has been conflicting [17].

An additional uncommon sequela in this patient was large volume hemoperitoneum discovered at the time of delivery. Hemoperitoneum stemming from a dysgerminoma, such as was observed in this case, has yet to be described in the literature. However, clinically significant hemorrhage from other adnexal masses has been described [16, 18], usually corpus luteal or hemorrhagic cysts. Symptoms that were noted earlier in this patient’s course, such as her upper abdominal pain, as well as laboratory findings such as her anemia and elevated LDH, are plausibly related to her hemorrhage. However, at the time these findings were identified, they were attributed to more common intrapartum conditions, namely HELLP syndrome in this case. During surgical exploration, the source of bleeding was identified as a ruptured vascular pedicle supplying the tumor. While very rare, we encourage readers managing pregnant patients with known adnexal masses to keep hemoperitoneum as part of their differential diagnosis in the event of signs or symptoms of anemia, acute abdomen, or hemodynamic instability.

Dysgerminomas are known to be rapidly growing, and there have been several reports documenting the development of large ovarian germ cell tumors during pregnancy in women with normal first trimester physical exams [2, 8, 19]. It is postulated that the dysgerminoma in our patient was hormone sensitive and grew exponentially in her later second and early third trimester. Hormone sensitivity of dysgerminomas has been documented in translational studies [20].

Ideally, management of suspected adnexal masses in pregnancy includes observation with surveillance via ultrasound at every trimester and management post-delivery. Surgical intervention should be strongly considered if there are acute complications such as torsion or rupture or if there is a 30–50% size increase at any time during the pregnancy [4, 5, 12]. Conservative surgical management for most malignant ovarian germ cell tumors diagnosed during pregnancy should be considered as the proper initial treatment [4, 5, 15, 19]. Surgical intervention of any kind in pregnancy is preferred between 14 and 22 weeks GA as it avoids the periods of greatest risk of drug induced teratogenicity and thus spontaneous fetal loss or intrinsic fetal abnormalities [5]. Conversely, a surgery in the mid-second or third trimester is technically more difficult and carries higher risk for adverse obstetrical outcomes [1, 4]. It is important to note that in cases where there is a high index of suspicion for a large pelvic mass or malignancy, a vertical midline incision should be created to allow for a complete exploration of the abdomen and contralateral ovary [3]. A Pfannenstiel technique was performed in this case because, at the time of incision, the diagnosis of a pelvic mass was still uncertain and provider comfort and urgency of delivery were in favor of a more typical surgical approach. Unfortunately, this decision limited visualization of the abdominal cavity and the ability to assess the omentum completely.

Overall, a majority of woman diagnosed with ovarian tumors during pregnancy have favorable results with low grade or early stage disease. It is appropriate to consider fertility sparing surgery in these young women with conservation of the contralateral ovary. Overall five-year survival rates approach 96% if the tumor is confined to the ovary [5, 8, 19]. This patient had follow-up CT scans showing complete resolution of disease, and her LDH rapidly normalized [9]. Although successful pregnancy with no fetal compromise was noted in this case, fetal demise has been reported in 25% of cases [13–15].

This case demonstrates that dysgerminomas may develop de novo in pregnancy and be an unexpected cause of labor dystocia or other abnormal symptoms. Although rare, adnexal masses should be part of a broad differential diagnosis when faced with abnormal labor progress and symptoms. Prompt identification can lead to appropriate management with an overall favorable outcome.

## Data Availability

Not applicable.

## Abbreviations

AFP: Alpha fetoprotein
FHT: Fetal heart tracing
GA: Gestational age
GCT: Germ cell tumor
HELLP: Hemolysis, elevated liver enzymes, low platelets
IV: Intravenous
LDH: Lactate dehydrogenase
PRBC: Packed red blood cells
TAUS: Transabdominal ultrasound
UTI: Urinary tract infection
